# How Does Personality Trait Affect Online Financial Service Use of College Students in China?

**DOI:** 10.3389/fpsyg.2022.847335

**Published:** 2022-06-24

**Authors:** Xiuyuan Gong, Xiaofeng Zheng, Qinqin Li

**Affiliations:** ^1^School of Business, Nanjing Normal University, Nanjing, China; ^2^Zhejiang Development and Planning Institute, Hangzhou, China; ^3^Jiangsu Key Laboratory of Modern Logistics, School of Marketing and Logistics Management, Nanjing University of Finance and Economics, Nanjing, China

**Keywords:** information technology, online financial service, materialism, attitude, college student, China

## Abstract

Online financial service is an essential part of consumption services provided by companies in modern society. It is vital to figure out the underlying mechanisms that influence online financial service use of college students in China, which is seldom explored. Drawing on the theory of planned behavior (TPB), this study explores the effect of personality traits (i.e., materialism) and its joint effect with attitude on online financial service use of college students. Moreover, we examined the interaction effects of key variables in TPB in the context of online financial services. The results indicated that the materialism value of Chinese college students has no direct effect on their intention to use online financial services but exerts an indirect effect through their attitude toward online financial services. College students' attitudes and perceived behavioral control are associated with their subjective norms, and in turn, affect their use intention of online financial services. In addition, perceived risk and perceived usefulness of online financial services also affect use intention through attitude and perceived behavioral control. The discussion of key findings, implications, and conclusions are provided.

## Introduction

Recently, the use of online financial services for consumption behavior has become a popular manner in China. The prevalence of online financial service benefits from the rapid growth of information technology and e-commerce such as online shopping (Ma and Lu, 2018). People can use financial services through different platforms such as *Huabei* provided by Alibaba Group or *Baitiao* provided by JD Company and conduct pre-consumption activities. A survey of Mckinsey has reported that China is the largest consumer financial market in the world (Hao et al., 2019; Liu and Zhang, 2021). The penetration rate of credit products among Chinese youngsters is 86.6%, and the use rate of online financial services is 61%, which is higher than the 46% use rate of credit cards (Nielsen, 2019). In China, female students spend a great proportion of living expenses on clothing, shoes, hats, make-up, and daily necessities, while male students prefer to pay money for electronic products or personal habits (iResearch, 2018; Luan, 2021). College students' decision on online financial service use has aroused the attention of the whole society. On the one hand, the online financial service is useful to college students in China. As China Banking Regulatory Commission (CBRC) suspended the issue of commercial bank credit cards to college students in 2009, which constrained the credit card use of college students, more and more students transferred to online financial services. The functions of online financial services are similar to that of offline bank credit cards, but they are more covenant-lite. On the other hand, online financial services such as online consumer credit may trigger several problems in college students' financial behavior, which has aroused the attention of society. For instance, 10.4% of college students have resulted in compulsive buying behavior because of the use of online consumer credit (He et al., 2018), and college students encountered financial difficulties may raise several health problems such as depression, anxiety, self-harm, attempted suicide, and somatic health complaints (Bøe et al., 2021). More seriously, some appalling incidents related to the granting of credit to undergraduates have occurred, raising broad concerns about the use of online financial services. In line with these points, an exploration on the underlying mechanisms of online financial service use of college student is important and necessary to researchers and practitioners.

The online financial service use of college students has become the focus of numerous studies. In prior studies, scholars have examined that the use or continue use intention of online financial services among college students may be affected by various factors. For instance, by collecting data from 126 respondents, Pi et al. (2012) highlighted the influential effect of website trust on the intention to continuous adoption of online financial services. Transaction security, prior Internet experience, website and company awareness, and navigation functionality positively affect cognitive trust, thus affecting the intention of continuous adoption directly or indirectly through effective trust. In the U.S. higher education markets, students who take more educational debt from for-profit institutions are likely to default on their student loans (Armona et al., 2022). The borrowers of federal loans are more likely to be female, unmarried, and lower-income, and the defaulters are more likely to be male, first-generation college, and lower-income. Compared with college students in the United States, college students in China may have lower levels of financial self-confidence and financial wellbeing (Norvilitis and Mao, 2013). The objective and subjective financial literacy of Chinese youngsters have direct negative effects on risky credit behavior such as risky paying or risky borrowing, besides, both of them can indirectly influence risky credit behavior through financial self-efficacy (Liu and Zhang, 2021). The cultivation path of college financial literacy involves three aspects, namely, establishing financial information security awareness, cultivating financial risk prevention awareness, and improving financial discrimination ability (Yida et al., 2021). Moreover, personality trait is considered to be related to the accumulation of debt and college students' financial future. For instance, Norvilitis et al. (2006) postulated that personality characteristics such as self-esteem or materialism can play a role in offline credit-card debt of college students. Similarly, Noh (2022) investigated a sample of 193 undergraduate and graduate students at a Midwestern university in the U.S. and proved that college students' financial behavior is influenced by their self-esteem, financial attitude, and parental financial teaching. To conclude, most of the previous studies focus on the demographic distribution (e.g., gender, income, area, and educational level), number of credit cards, financial literacy, delay of gratification in affecting financial service use of college students, or health problems (e.g., mental health or physical health) of college students who suffer from financial debt or difficulties, there is still scant research on investigating the effects of personality traits such as materialism on college students' intention to use online financial service, and how they balance the usefulness of online financial service and the potential risks may be encountered.

It is noticed that college students' decision-making process is related to various complicated factors such as their perception and their personality (Ju et al., 2019; Miller, 2019). They may take possessions of material goods as a symbol of status or social standing, which is closely related to self-identity (Belk, 1985), thus personality factors such as materialism may have a great impact on their subsequent behavior. Following this logic, this study takes materialism as an essential personality trait that may engender the online financial use of Chinese youngsters. Besides, individual perceptions such as perceived risk and perceived usefulness are two important perceptions that induce people to balance the gain and loss, thus likely to have effects on behavioral intention and decision-making (Yang et al., 2015; Hansen et al., 2018; Hwang and Choe, 2020). Therefore, this study is justified on the basis of the characteristics of college students and their online financial service use by applying the theory of planned behavior (TPB), which is one of the most established theoretical framework in the analysis of behavioral intention (Gollwitzer and Sheeran, 2006), and develops a systematic model to explain the joint effects of driving forces (materialism, perceived risk, perceived usefulness, attitude, subjective norm, and perceived behavioral control) in influencing online financial service use of college students in China.

## Theoretical Background and Hypotheses

### The Theory of Planned Behavior (TPB)

The TPB is a psychological theory that connects beliefs to intention, it is an extension of the theory of reasoned action (TRA) (Ajzen and Fishbein, 1980), and it becomes one of the most influential and popular theoretical frameworks in predicting and explaining behavioral intention or behavior of human (Pavlou and Fygenson, 2006). TPB postulated that behavioral intention is an immediate antecedent of actual human behavior (Ajzen, 2002), which relates to an individual's intention to engage in a particular behavior (Palau-Saumell et al., 2021). According to this theory, human behavior is guided by three different beliefs: attitude toward the behavior, subjective norm, and perceived behavioral control (Ajzen, 2002), with each predictor weighted for its importance in relation to the behavior and population of interest. Attitude refers to a person's overall evaluation of performing a behavior, which is determined by a range of underlying beliefs, for instance, beliefs about the likely consequences or other attributes of the behavior (behavioral beliefs). Subjective norm captures a person's perception of whether important others approve of that the person performing a behavior, and it refers to beliefs about the normative expectations of other people (normative beliefs). Perceived behavioral control is the added variable to TRA. In the TPB, it refers generally to people's expectations regarding the degree to which they are capable of performing a given behavior, the extent to which they have the requisite resources (e.g., skills, time, money, and cooperation with others), and believe that they can overcome whatever obstacles they may encounter (Ajzen, 2002, p. 676–677), thus this component is related to beliefs about the presence of factors that may further or hinder the performance of the behavior (control beliefs). In this analysis, the intention is defined as college students' inclination to use online financial services, attitude is defined as college students' beliefs and personal conviction about using online financial services, subjective norm is defined as college students' perception of what their salient referents would think about use of online financial service, and perceived behavioral control refers to the perception of college students about their confidence and capability to control use or not to use online financial service, thus it involves controllability and self-efficacy (Ajzen, 2002). Previous research has identified the positive relationships between attitude, subjective norm, perceived behavioral control, and travel intention of residents in Spanish territory affected by the COVID-19 pandemic (Sánchez-Cañizares et al., 2021). Accordingly, we proposed the following hypotheses:

*H1*: *Attitude toward using online financial services positively influences college students' intention to use online financial services*.
*H2*: *Subjective norm positively influences college students' intention to use online financial services*.
*H3*: *Perceived behavioral control positively influences college students' intention to use online financial services*.

Besides, subjective norm may play a role in affecting college students' perceived behavioral control and attitude toward online financial service use. The social environment is important to a person, and its attributes also have influences on the attributes of the person (Eagly, 2009). Regarding college students, they are likely to be influenced by others' expectations and to comply with others' opinions when they form their own attitudes, especially the opinions of their salient referents such as parents or good friends. Previous research also pointed out the avenue through which attitude functions as a mediator between subjective norm and behavioral intention of consumers in the restaurant industry (Kim et al., 2013). The following hypothesis is proposed:

*H4*: *Subjective norm has a positive impact on college students' attitude toward using online financial services*.

In addition, college students are dependent on their parents in many aspects, and they also rely on their good friends in campus life. In this regard, subjective norm is likely to exert social pressure on college students, which may affect students' perception and evaluations of their capability and controllability of using online financial services. Therefore, we conjectured that subjective norm has positive impacts on college students' attitudes and perceived behavioral control of online financial service use. Thus, we proposed the following hypothesis:

*H5*: *Subjective norm has a positive impact on college students' perceived behavioral control about using online financial services*.

### Perceived Risk of Online Financial Service

The concept of risk is first introduced by Knight (1948), who posited that risk is a critical component of economic activity. Risk has been examined in a range of disciplines, including marketing (Stone and Grønhaug, 1993), tourism (Steiger et al., 2019), psychology (Iverson et al., 2019), and sociology (Keddell and Hyslop, 2019). Bauer (1960) introduced the variable of “risk” to the field of marketing and described the notion of perceived risk in buying behavior. It is found that “consumer behavior involves risk in the sense that any action of a consumer will produce consequences which he cannot anticipate with anything approximating certainty, and some of which at least are likely to be unpleasant” (Bauer, 1960, p. 21). In other words, perceived risk is a subjective perception concerning the uncertainty and negative consequences of a behavior (Palau-Saumell et al., 2021) such as the potential loss of conducting a behavior (Dholakia, 2001). The uncertainty of using online financial services is also related to the risk of potential loss including personal information abuse or unnecessary expenses due to improper use. In an investigation of the airline industry, Han et al. (2019) found that a high level of perceive risk on electric airplanes is negatively related to consumers' attitude toward electric airplanes. Similarly, Sánchez-Cañizares et al. (2021) also proved the negative relationship between perceived risk of tourists and their attitude to travel. Following these arguments, we proposed the following hypothesis:

*H6*: *Perceived risk negatively influences college students' attitudes toward using online financial services*.

Previous research has shown that risk assessment is crucial to explore undergraduate loan behavior (Luan, 2021), and perceived risk is negatively related to perceived behavioral control in the COVID-19 situation (Sánchez-Cañizares et al., 2021). For Chinese college students, the perceived risk of online financial services includes potential financial fraud, credit defaults, and other pitfalls (Liu and Zhang, 2021). It is anticipated that the greater the perception of potential negative consequences arising from using online financial services, the less control the individual would feel he/she has over that behavior. Thus, the following hypothesis is proposed:

*H7*: *Perceived risk negatively influences college students' perceived behavioral control about using online financial services*.

### Perceived Usefulness of Online Financial Service

Perceived usefulness is one of the key determinants of the technology acceptance model (TAM), which is one of the most cited conceptual frameworks to predict the acceptance and use of new information technology. According to the TAM framework, perceived usefulness, perceived ease of use, and attitude toward use are three essential factors that explain a user's motivation in accepting information technology (Davis, 1985). Perceived usefulness is defined as “the degree to which a person believes that use of the system will enhance his or her performance” (Davis, 1989, p. 320), and it is described as an important predictor of users' attitudes toward using information technology. In previous literature, perceived usefulness has also been proven to have a positive effect on attitude in various contexts such as digital learning technology (Sayaf et al., 2022) or electronic banking (Jahangir and Begum, 2008). This study defines that perceived usefulness is the degree to which a college student believes that using online financial services would be beneficial to his/her life and proposes that perceived usefulness is positively associated with attitude toward using online financial services.

*H8*: *Perceived usefulness positively influences college students' attitudes toward using online financial services*.

Perceived usefulness is also likely to affect the perceived behavioral control of college students on online financial services. It is posited that perceived usefulness is positively related to trust beliefs (Song, 2007; Bilgihan et al., 2016), and trust beliefs positively affect controllability (Pavlou and Fygenson, 2006), which is one of the reflections of perceived behavioral control (Ajzen, 2002). In China, college students can immediately obtain credit after registering on online financial service platforms, conduct pre-consumption, and repay it in installments (Liu and Zhang, 2021), thus online financial service is useful to college students. The fewer restrictions and useful attributes of online financial services exhibit salient advantages compared with traditional financial services, which may increase the feeling of control by using online financial services. Therefore, the following hypothesis is conjectured:

*H9*: *Perceived usefulness positively influences college students' perceived behavioral control about using online financial services*.

### Materialism of College Students

Materialism refers to a belief that material possessions can signal success, bringing happiness, which are important goals of one's life (Richins and Dawson, 1992; Dittmar, 2005; Donnelly et al., 2013). Differing from others who buy goods for the benefits they provide, some people place a disproportionate emphasis on goods to meet their demands and value acquisition as a way to achieve important life goals, which is labeled as materialists. It is found that these people devote considerable time to planning purchases and may endure discomforts to acquire their desired objects. People with a high level of materialism more likely perceive themselves as “spenders” (Garð*arsdóttir and Dittmar, 2012). They often buy material goods for the purpose of transforming their lives or pursuing the “good life” (Scott and Alwin, 1998; Mishel et al., 2005) or desire to achieve a certain social status and prestige (Limbu et al., 2012; Kim and Jang, 2014), which may lead to excessive spending and other financially risky behaviors such as bankruptcies and gambling (Richins, 2011). Richins and Dawson (1992) conceptualized materialism as a consumer value and test it at an individual level.

Many researchers have recognized the credit card use/misuse of college students and have pointed out that factors such as materialism, age, gender, and parental influence are associated with credit card debt. Among these factors, materialism can enhance commitment, trust, and use, but impedes preeminent balance management (Limbu et al., 2012). Pinto et al. (2000) conducted research on American college students and found that college students with high materialism use credit cards to purchase more clothing and gifts than the low materialism group, thus placing more value on the “show” or social impression of material possessions. Tascioglu et al. (2017) explored the moderating effect of materialism on the link between the status motivation of college students and their perceptions of social sustainability. Peltier et al. (2016) examined the effect of materialism on student credit card debt. They argued that materialism and impulsivity are related to self-control lapses, which are the results of irrational decision-making.

In prior studies, Pradhan et al. (2018) provided evidence that materialism exerts an effect on individual credit card use, but they did not consider the joint effects of materialism and attitude. Similarly, Zainudin et al. (2019) proved that materialism and attitude are separate determinants of credit card misuse among Generation Y in Malaysia. Garð*arsdóttir and Dittmar (2012) posited that high levels of materialism give rise to buying motives. People with high materialistic values have more positive attitudes toward borrowing and are willing to carry heavier debt loads (Watson, 1998, 2003; Ponchio and Aranha, 2008). For Chinese college students, Liu and Zhang (2021) pointed out that college students are the main users of online financial services such as online consumer credit, and they are adept at shopping online for diversified consumption demands (e.g., fashion, entertainment, and social networking). The materialism of college students is likely to determine their attitude toward using online financial services. The following hypothesis is therefore proposed:

*H10*: *College students with a high level of materialism are more likely to have a positive attitude toward using online financial services*.

Prior studies revealed that a high level of materialism is related to consumers' intentions to adopt direct and indirect pro-environmental behaviors (PEBs) (Alzubaidi et al., 2021) and purchase intention of sustainable luxury goods (Kaur et al., 2022). In China, college students also exhibit different levels of materialism. For instance, female students may spend a proportion of living expenses on clothing, shoes, hats, make-up, and daily necessities, while male students usually pay money for electronic products (including game devices and equipment) (Luan, 2021). As a result, college students with a high level of materialism may take online financial services as a mechanism through which they can achieve material goods or enhance social status, which leads to more favorable attitudes toward online financial service use. Consequently, the following hypothesis is proposed:

*H11*: *College students with a high level of materialism are more likely to use online financial services*.

**Figure 1:**
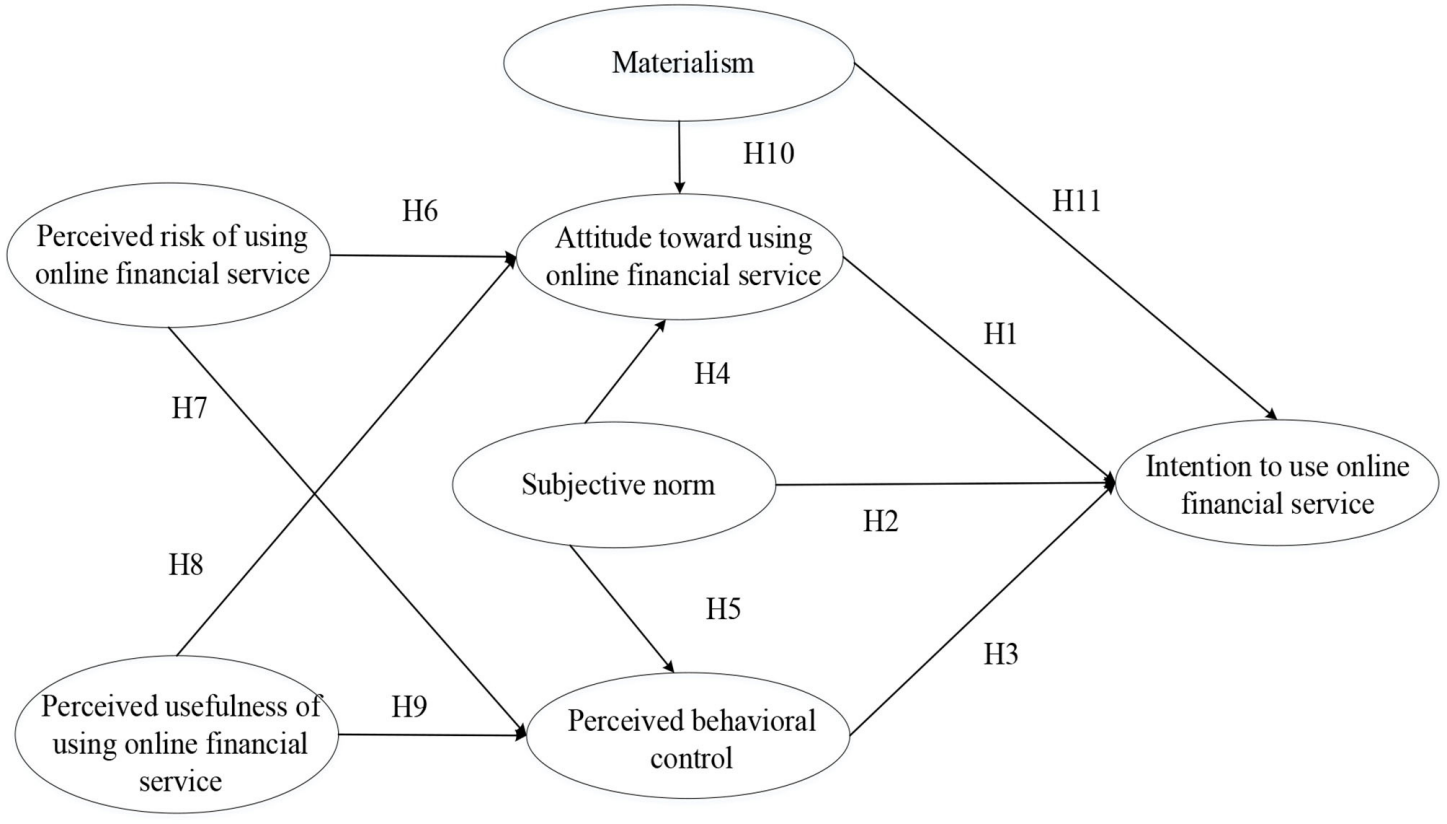
The research model.

In summary, materialism is conjectured to be a crucial personal trait that affects college students' intention to use online financial services. Perceived risk and perceived usefulness are perceptions that may affect college students' intention through their attitude and perceived behavioral intention, as well as subjective norms. Figure 1 summarizes these hypothesized relationships between these variables of the conceptual model.

## Methodology

### Data Collection

This study collected data from college students in universities in Nanjing, the capital of Jiangsu Province. Nanjing is one of the first-tier cities in China,^1^ and it has abundant educational resources that attract a lot of college students. In addition, Jiangsu belongs to the Yangtze River Delta of China, with an extremely prosperous Internet financial industry. Thus, it was chosen to collect samples to represent college students in China. We adopted a convenience sampling method and collected data from college students through an online survey. This sampling method would help us to approach samples of college students and thus improve the accuracy and reliability of the results. Finally, we had 310 valid responses. As Comrey (1988) proposed that “A sample size of 200 is reasonably good for ordinary factor-analytic work with 40 or fewer variables”, the number of items in our questionnaire is 20; therefore, it is appropriate to use 200+ samples to explain the model. Table 1 shows the demographic information of respondents. Among 310 respondents, 196 are women and 116 are men. Notably, 94.5% of respondents were aged 18–25 (*M* = 20), which is similar to iResearch (2018) and Nielsen (2019). A total of 47.1% of respondents are sophomores, and 21.3% of respondents are freshmen. For the monthly budget, 58.7% of respondents have 1,500–2,000 yuan, and only 1.6% of respondents have 4,000 yuan or more money. The main source of money is from parents (94.8%), and other sources of money are from part-time jobs (23.2%) or scholarships (21.3%). In total, only 22.2% of respondents thought that their pocket money is more than their needs. Most of the respondents have pre-consumption experience (69%).

**Table 1:** Demographics of respondents.

**Demographics**		**Frequency**	**Percentage**	**Mean**	**Standard**
			**(%)**		**deviation**
Gender	Female	196	63.2	1.37	0.483
	Male	114	36.8		
Age	<18 years	5	1.6	2.02	0.233
	18–25 years	293	94.5		
	>25 years	12	3.9		
Grade	Freshman	66	21.3	2.35	1.175
	Sophomore	146	47.1		
	Junior	54	17.4		
	Senior	10	3.2		
	Graduate student	34	11		
Monthly budget (yuan)	<1,500	92	29.7	1.84	0.660
	1,500–2,000	182	58.7		
	2,000–4,000	31	10		
	> 4,000	5	1.6		
Pocket money	More than I need	69	22.2	1.99	0.658
	Enough	176	56.8		
	Less than I need	65	21		
Pre-consumption experience	Yes, I had	214	69	1.31	0.463
	No, I hadn't	96	31		

### Measures

For the measurement of variables in TPB, we adopted the mature scales from previous studies to guarantee reliability and validity, and modified them to fit the study context. All the items were rated by respondents on 5-point Likert scales, anchoring from 1 (strongly disagree) to 5 (strongly agree). For instance, attitude was measured with a two-item scale that was adapted from Elliott et al. (2003) and Cordano and Frieze (2000), an example of items was “I am inclined to support the use of online financial service.” By analyzing attitude items in previous literature, Piazza (1980) pointed out that the use of a single item as the measure of attitude is not optimal but reasonable, and “in any case, it is usually possible to obtain at least two or three items that can be combined into a scale of a given attitude” (Piazza, 1980, p. 602). A prior study also use two items to measure attitude (Lu et al., 2005). In line with these arguments, the measurement of attitude in this study is rationalized.

Similarly, we used two items to measure subjective norm, which were also adapted from Elliott et al. (2003) and Cordano and Frieze (2000), an example of items is “People who are important to me (e.g., my family and boyfriend/girlfriend) would approve me to use online financial service.” This is consistent with previous studies, which measure subjective norm with two items to describe the family or friends/colleagues' opinions when individuals make the decision to travel in the COVID-19 pandemic (Sánchez-Cañizares et al., 2021) or planning to use computer technology (Teo and Lee, 2010).

Perceived behavioral control can be measured by asking direct questions about the capability to perform a behavior or indirectly on the basis of beliefs about the ability to deal with specific inhibiting or facilitating factors (Ajzen, 2002, p. 668). Following this suggestion, we measured perceived behavioral control with two items (i.e., When I want to use online financial services, I can find them on the Internet, and Whether or not to use online financial services is entirely up to me) to reflect self-efficacy and controllability. The measures were adapted from Cordano and Frieze (2000) and Quintal et al. (2010), which was consistent with Sánchez-Cañizares et al. (2021).

Behavioral intention captures how people are willing to or intend to perform a behavior. Similar to previous studies (Lu et al., 2005; Teo et al., 2009; Teo and Lee, 2010; Hsu, 2012; Palau-Saumell et al., 2021), we used two items to measure behavioral intention. The scales of intention to use online financial services were developed by Elliott et al. (2003), to measure the intention of college students to use online financial service and their likelihood to use online financial services. A sample item was “I intend to use online financial service frequently.” The measures were also consistent with Wang et al. (2006), which analyze data collected from 258 users to predict consumer intention to use mobile services.

Perceived risk is a perception that examined how college students evaluate the consequences that they use online financial services. This construct was measured using four items from Wei et al. (2016) and Terpstra (2011) to investigate college students' conceptions about the threats to personal safety, property, family, and daily life. Examples of items from the scale were “I'm worried about incurring property damage due to the loss, theft, and forgery of personal information” or “I'm worried that online financial service would cause a threat to my family.”

Perceived usefulness is an important factor in TAM, which is proposed by Davis (1989). It refers to the beliefs of Chinese college students that using online financial services would enhance their performance such as shopping online. In this study, we mainly adopted the perceived usefulness scale from Davis (1989). Perceived usefulness was assessed using five items from Davis (1989) and evaluated from five aspects (i.e., work more quickly, performance, increase productivity, effectiveness, and make the job easier and useful) (Davis, 1989, p. 331). An example of items from the scale was “Online financial service is useful to me.”

Materialism is a “set of centrally held beliefs about the importance of possessions in one's life” (Richins and Dawson, 1992, p. 308). For the measurement of materialism, the materialism scale of Richins and Dawson (1992) is the most widely used instrument to measure individual materialism (Shrum et al., 2013; Srikant, 2013). According to Richins and Dawson (1992), materialism can be assessed from three aspects: success (a belief that an individual's success depends on their possession of material goods), happiness (a belief that owing the desirable possessions entails wellbeing), and acquisition centrality (the importance materialists attach to the acquisition and possessions of material goods central to their lives). This study also adapted from Richins and Dawson (1992) materialism scale and modified by the research context. Finally, a four-item scale of materialism was used, and an example of items was “I admire people who own expensive homes, cars, and clothes” or “It sometimes bothers me quite a bit that I can't afford to buy all the things I'd like.”

## Results

### Measurement Model

The measurement model was examined by assessing its reliability and validity. The reliabilities of constructs were assessed *via* Cronbach's alpha values, composite reliability (CR), and factor loadings (Bagozzi and Yi, 1988; Hair et al., 2011). Table 2 shows that the values of Cronbach's alpha range from 0.79 to 0.92, which is higher than the recommended value of 0.70. The CR of each construct ranged from 0.90 to 0.95, thus fulfilling the benchmark value of 0.70. All the factor loadings were higher than the threshold value of 0.70. The outcome demonstrated that the reliability is acceptable. Convergent validity is established when the average variance extracted (AVE) for each construct exceeds 0.50 (Fornell and Larcker, 1981; Hair et al., 2011). As shown in Table 2, all the AVE for each construct met the requirements. Discriminant validity is achieved when the square roots of AVE for each construct are higher than the correlation matrix of the constructs (Hair et al., 2011). Table 3 shows that the discriminant validity is satisfactory.

**Table 2:** Results of confirmatory factor analysis.

**Constructs**	**Items**	**Loading**	**Cronbach's**	**Composite**	**AVE**
			**alpha value**	**reliability**	
Attitude (ATT)	ATT1	0.95	0.89	0.95	0.90
	ATT2	0.95			
Subjective norm (SN)	SN1	0.92	0.84	0.93	0.86
	SN2	0.94			
Perceived behavioral control (PBC)	PBC1	0.90	0.79	0.90	0.82
	PBC2	0.92			
Perceived risk (PR)	PR1	0.86	0.89	0.92	0.75
	PR2	0.90			
	PR3	0.77			
	PR4	0.91			
Perceived usefulness (PU)	PU1	0.89	0.92	0.94	0.81
	PU2	0.91			
	PU3	0.90			
	PU4	0.90			
Materialism (MAT)	MAT1	0.81	0.84	0.90	0.68
	MAT2	0.85			
	MAT3	0.86			
	MAT4	0.79			
Intention to use online financial service (InOFS)	InOFS1	0.95	0.89	0.95	0.90
	InOFS2	0.95			

**Table 3:** The correlations between constructs.

**Constructs**	**PR**	**PU**	**ATT**	**SN**	**PBC**	**MAT**	**InOFS**
Perceived risk (PR)	**0.86**						
Perceived usefulness (PU)	0.30	**0.90**					
Attitude (ATT)	0.01	0.60	**0.95**				
Subjective norm (SN)	0.05	0.52	0.68	**0.93**			
Perceived behavioral control (PBC)	0.21	0.49	0.57	0.45	**0.91**		
Materialism (MAT)	0.22	0.47	0.60	0.50	0.43	**0.83**	
Intention to use online financial service (InOFS)	0.05	0.59	0.64	0.58	0.47	0.44	**0.95**

*The diagonal elements (bold values) represent the square roots of average variance extracted (AVE) for each construct*.

This study used the global goodness-of-fit (GoF) to assess the GoF of models, which is consistent with Chinomona and Sandada (2013). This measure considered both the measurement model and structural model. Finally, the value of GoF was 0.62, which exceeded the recommended threshold of GoF > 0.36 (Wetzels et al., 2009). By following Podsakoff et al. (2003), we also checked the common method bias (CMB) by conducting Harman's single-factor test. When one general factor can explain the majority of the covariance of the variables, CMB will be a problem. The rotated principal component factor analysis showed 18.42% of the total variance, thus rejecting the probability of one general factor. Besides, we assessed multicollinearity and found that the variance inflation factors range from 1.22 to 2.93, which is lower than the accepted cutoff of 5 (Hair et al., 2012). Thus, multicollinearity was not a threat to this study.

### Structural Model

**Figure 2:**
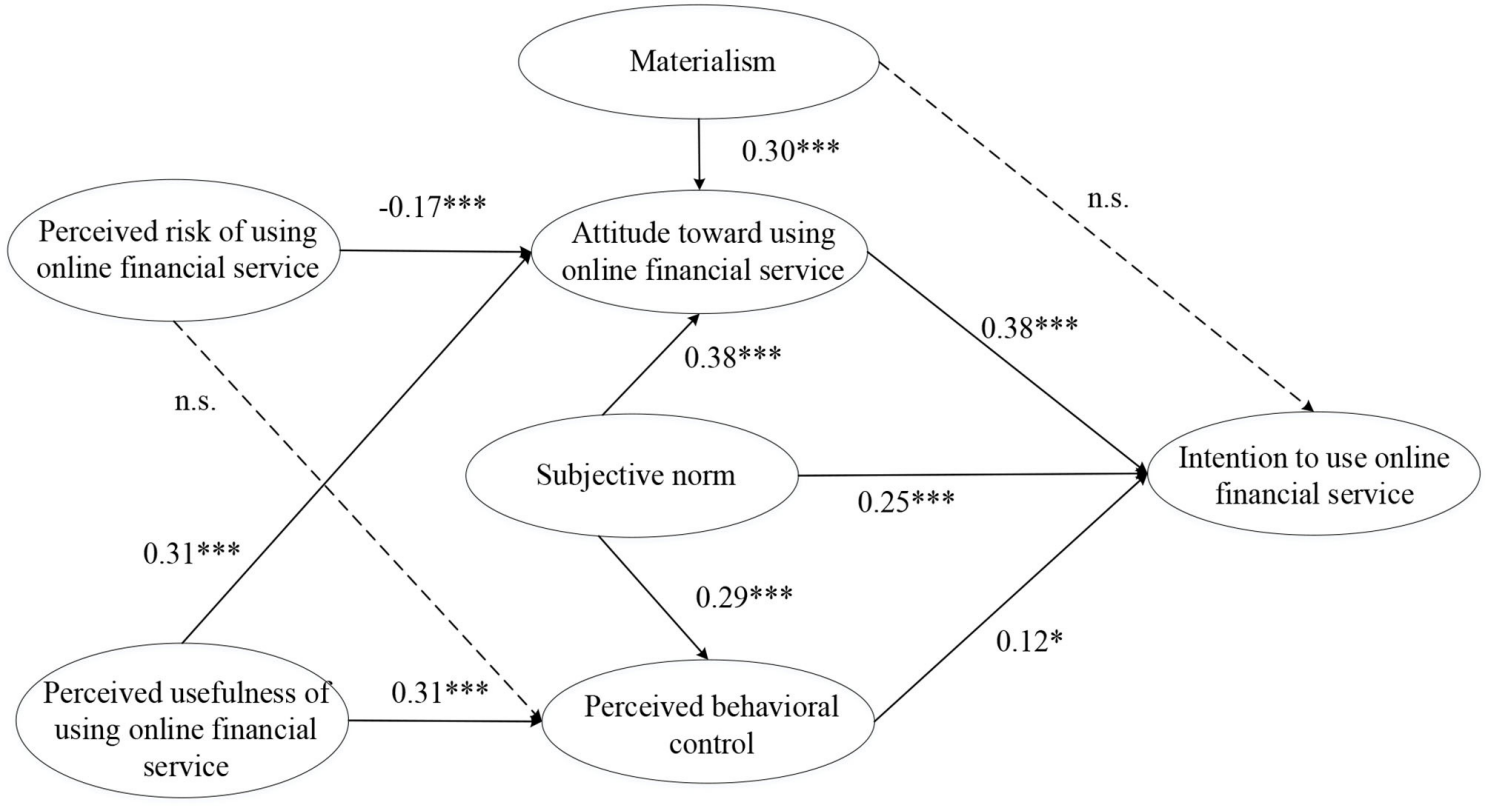
The structural model with hypothesis testing. ***p < 0.001, *p < 0.05.

This study utilizes partial least square (PLS) for path analysis based on its advantages. PLS is good at conducting exploratory studies and is able to estimate the indicator loadings and the causal relationships between constructs (Fornell and Bookstein, 1982; Hair et al., 2011). Compared with the covariance-based structural equation modeling (SEM) approach, PLS is more appropriate to analyze the small samples (Hair et al., 2011). Thus, it is suitable to use PLS in this study. The analytical results are shown in Figure 2. For the direct effects of intention to use online financial service, students' attitude positively influences their intention to use online financial service (β = 0.38, *p* < 0.001). Subjective norm has a significant positive effect on students' intention to use online financial service (β = 0.25, *p* < 0.001). Likely, perceived behavioral control also has a positive effect on the intention to use online financial services (β = 0.12, *p* < 0.05). Thus, H1, H2, and H3 are supported. However, the direct effect of materialism on intention to use online financial services is insignificant (β = 0.04, *p* > 0.05), thus H11 is not supported. For the relationships between other constructs, we found that subjective norm has a significantly positive effect on attitude (β = 0.38, *p* < 0.001), providing support for H4. In contrast, perceived risk has a negative effect on attitude (β = −0.17, *p* < 0.001), supporting H6. However, the impact of perceived risk on perceived behavioral control is insignificant (β = 0.10, *p* > 0.05), thus H7 is not supported. Besides, subjective norm has a positive effect on perceived behavioral control (β = 0.29, *p* < 0.001). Similarly, perceived usefulness positively influences attitude (β = 0.31, *p* < 0.001) and perceived behavioral control (β = 0.31, *p* < 0.001), respectively. As a result, H5, H8, and H9 are supported. The analysis showed that *R*^2^-values of dependent variables range from 0.30 to 0.62, accounting for a considerable proportion of the variance in the research model.

### Mediation Analyses

By following Hajli et al. (2017), we used bootstrapping technique to test the mediation effects. Most indirect effects were bootstrapped 1,000 times to estimate the 95% confidence intervals (CIs), except the indirect effects of perceived risk. The mediation effects are considered to be significant if the CI did not include the value of zero. Moreover, the full mediation has occurred if the β is insignificant after controlling for the mediator (Baron and Kenny, 1986; Preacher and Hayes, 2004). As shown in Table 4, attitude fully mediates the relationship between materialism and intention to use online financial service (β = 0.11, *p* > 0.05; CI = 0.34, 0.55). Due to the absence of 0 in the CI, attitude partially mediates the relationship between perceived usefulness and intention to use online financial service (CI = 0.23, 0.39) and between subjective norm and intention to use online financial service (CI = 0.30, 0.52). In addition, perceived behavioral control partially mediates the relationship between perceived usefulness and intention to use online financial service (CI = 0.07, 0.19) and between subjective norm and intention to use online financial service (CI = 0.08, 0.21).

**Table 4:** Mediation analyses.

**Path**	**Path *c* (X → Y)**	**Path *a* (X → M)**	**Path *b* (M → Y_**.X**_)**	**Path *c'* (X → Y_**.M**_)**	**Indirect effect** **(95% confidence interval)**			**Type**
	**B**	**B**	**B**	**B**	**Effect**	**Lower**	**Upper**	
Materialism → Attitude → Intention to use online financial service	0.55^***^	0.57^***^	0.78^***^	0.11	0.45^†^	0.34	0.55	Full mediation
Perceived usefulness → Attitude → Intention to use online financial service	0.67^***^	0.51^***^	0.60^***^	0.36^***^	0.31^†^	0.23	0.39	Partial mediation
Subjective norm → Attitude → Intention to use online financial service	0.74^***^	0.65^***^	0.62^***^	0.33^***^	0.41^†^	0.30	0.52	Partial mediation
Perceived usefulness → Perceived behavioral control → Intention to use online financial service	0.67^***^	0.45^***^	0.30^***^	0.54^***^	0.13^†^	0.07	0.19	Partial mediation
Subjective norm → perceived behavioral control → Intention to use online financial service	0.74^***^	0.45^***^	0.33^***^	0.59^***^	0.15^†^	0.08	0.21	Partial mediation

*1,000 bootstrap samples with 95% confidence level*.

*** *p < 0.001*.

*Path c = relationship between IV and DV; Path a = relationship between IV and mediator; Path b = relationship between mediator and DV, controlling for IV; Path c' = direct effect of IV on DV, controlling for mediator*.

† *indicates the indirect effect is significant*.

## Discussion and Conclusion

### Discussion

This study proposes a conceptual model and predicts college students' intention to use online financial services in China. In particular, we investigated the effect of personality traits (i.e., materialism) on the use of online financial services among college students and examined an extended TPB model. The analytical results showed that most of the hypotheses are supported, thus validating the proposed model.

There are a number of key findings. First, college students' intention to use online financial services can be predicted by students' attitudes, subjective norms, and perceived behavioral control, which is consistent with the findings of Nie et al. (2020) in a different research context. In particular, attitude plays the most significant effect on online financial service use intention. The direct effect of materialism on the intention to use online financial services is insignificant, which may be explained by the mediating effect of attitude. From the results of mediation analyses, we can find that attitude fully mediates the relationship between materialism and intention to use online financial services. Therefore, materialism can only influence the intention to use online financial services through attitude in this research context. This finding is consistent with the study by Peltier et al. (2016) that materialism has an indirect effect on the credit card debt of college students. Moreover, the findings of this study indicated that attitude is also determined by subjective norm, perceived risk, and perceived usefulness, while perceived behavioral control is influenced by subjective norm and perceived usefulness. However, the effect of perceived risk on perceived behavioral control is insignificant. The possible reason is that college students may have insufficient risk recognition or limited risk knowledge because they are young and lack social experience. Besides, platforms such as *Huabei* and *Baitiao* will provide reasonable loans to students by evaluating their personal information such as savings and expenditure, which reduces the risk perception of college students in China. College students may have less bit of worry about the negative impacts of online financial services, but their worry cannot prevent their behavior of seeking or using online financial services. Thus, the finding revealed that perceived risk is not significantly associated with perceived behavioral control.

### Theoretical Implications

The analytical results provide several theoretical implications. First, this study investigates the effect of personality trait (i.e., materialism) and its joint effect with attitude on college students' intention to use online financial services in China, which enriches the study of materialism and online financial service. Materialism is regarded as an important personality trait in determining college students' intention to use online financial services in this study. Prior studies have explored the effects of materialism on behavioral intention, but they may not consider the joint effects of materialism with other factors such as attitude. For instance, Kaur et al. (2022) collected data from 229 respondents and examined the separate impacts of materialism and attitude on purchase intention of sustainable luxury products in India. This study explores the joint effects of materialism and attitude, thus contributing to related literature. Moreover, the findings revealed that the effect of materialism can be fully mediated by college students' attitudes. In other words, Chinese college students with a high level of materialism would display a higher level of intention to use online financial services through attitude toward behavior. This finding is consistent with prior studies that personality traits would affect individual financial behavior (Noh, 2022), purchase intention (Septiana and Qastharin), or repurchase intention (Chandra, 2021) through attitude toward behavior.

Second, this study considers the joint effects of key variables in TPB and contributes to TPB literature and online financial service studies. Most of the previous studies have explored the separate effects of attitude, subjective norm, and perceived behavioral control on behavioral intention (Teo and Lee, 2010; Palau-Saumell et al., 2021; Sánchez-Cañizares et al., 2021), and the underlying mechanisms that how key variables in TPB interact are seldom explored. This study confirms that subjective norm has a positive impact on attitude, which is in accordance with the arguments of Kim et al. (2013) that attitudinal and normative constructs in TPB are not as independent as predicted. A mediating effect identifies the impact consumers' subjective norm has on behavioral intention through attitude toward behavior. The finding is consistent with previous studies (Chang, 1998; Han et al., 2010). Similarly, a positive and significant path from the subjective norm to perceived behavioral control shows an explanatory power of intention. The implications are that the formation of attitude and perceived behavioral control, influenced by college students' important referents, such as family members, teachers, and friends, affects the formation of behavioral intention. In addition, the standardized coefficients and *t*-values indicate that the direct impact of attitude on intention to use online financial services is greater than perceived behavioral control and subjective norm.

Third, this study examines the effects of perceived risk and perceived usefulness on intention to use online financial services, which advances the related literature on college students' online financial service use in China. Both perceived risk and perceived usefulness are critical factors that influence behavior intention (Hansen et al., 2018). This study takes them into consideration and explores their indirect effects on college students' intention to use online financial services in China. Consistent with the argument of Baidoo and Natarajan (2021) that lenders' attitudes toward risk are essential in evaluating their lending decisions, the findings revealed that the perceived risk of college students has a significant effect on their attitude, which subsequently affects their online financial service use intention. Besides, the findings revealed that attitude partially mediates the relationship between perceived usefulness and intention to use online financial services, which is consistent with the finding of Davis (1989) that attitude did not fully mediate the perceived usefulness.

### Practical Implications

This study has valuable implications for practitioners. First, the results indicated that materialism is an important factor to influence online financial service use. For schools and teachers, it is critical to them to recognize college students' materialism level and be responsible to cultivate college students' positive value formation regarding material goods. For instance, they could educate college students that material possessions are not equal to success and happiness and explain what is the right way to get happiness and success. Apart from the common impressions about materialism (e.g., success and happiness) from material possessions, schools and teachers are able to guide college students to develop a correct understanding of online financial services.

Besides, the results showed that college students' attitudes, subjective norms, and perceived behavioral control are key drivers of their intention to use online financial services. For college students in China, their attitude is the most significant determinant of online financial service use intention. The formations of college students' attitudes and perceived behavioral control are influenced by their important referents, such as family members, teachers, and friends. In this regard, both parents and teachers should pay more attention on students' attitudes and perception formation about online financial services in daily life. As the important referents, their opinions, attitude, and behaviors are critical to college students' identification of online financial services and influence students' online financial service use through attitude and perceived behavioral control.

In addition, the findings suggested that perceived risk and perceived usefulness also influence students' intention of using online financial services indirectly. Regarding teachers and parents, their duties are still to educate college students in terms of the possible risks (e.g., high-interest borrowing, unnecessary expenses, credit abuse, credit damage, and defaulted loans) and usefulness (e.g., convenience of use, and easy and quick lending process) of online financial service from every aspect. Regarding online financial service providers, they need to take specific strategies for the service application of college students. Although increasing the perceived usefulness of online financial services will be a good approach for attracting students to use online financial services, service providers should highlight the potential risks of online financial services and offer related knowledge, such as interest charges, comprehensive loan repayment procedures, and other loan repayment issues, to show their ethics responsibility (Austin and Phillips, 2001) on college student groups. Moreover, service providers should improve their safety measures to reduce potential risks of online financial services concerning student users because college students are financially dependent and have insufficient knowledge about online financial services.

### Limitations and Future Research

Although this study provides new insights and contributes to the literature, it also has a few limitations. The first limitation is from a methodological perspective, as this study uses cross-sectional data from self-reported respondents, thus may lead to potential common method variance. To deal with this problem, we have conducted Harman's single-factor test by following Podsakoff et al. (2003). Future studies could also use multiple methods (e.g., longitudinal method) to collect data and better address the issue of common method variance arising from a single method of data collection. Second, materialism is evaluated from an individual perspective in this study. However, materialism can be also evaluated as a socio-cultural phenomenon (Srikant, 2013), thus future studies could evaluate the cultures of research context in which the majority of the people in the social value material objects and compare the oscillates between materialism and altruism in the context. Third, this study takes perceived risk as an overall construct, and future studies could examine the effects of different kinds of perceived risk (e.g., security risk, privacy risk, and social risk) on online financial service use of college students. The operationalization and measurements of the key variables in this study can also be different according to the specific research context. Fourth, this study considers several predictors such as materialism, perceived risk, and perceived usefulness, and examines their influence on intention to use online financial services. Future studies could examine other potential predictors (e.g., financial literacy, financial education, and prior consumption behavior) or considers more aspects on financial wellbeing such as financial vulnerability or financial hardship, which may induce interesting topics for future research.

## Data Availability Statement

The original contributions presented in the study are included in the article/supplementary material, further inquiries can be directed to the corresponding authors.

## Author Contributions

XG: investigation, formal analysis, validation, writing, and funding acquisition. XZ: conceptualization, methodology, and project administration. QL: data collection and visualization. All authors contributed to the article and approved the submitted version.

## Funding

This work described in this paper was supported by the National Natural Science Foundation of China (Project No. 72001114).

## Conflict of Interest

The authors declare that the research was conducted in the absence of any commercial or financial relationships that could be construed as a potential conflict of interest.

## Publisher's Note

All claims expressed in this article are solely those of the authors and do not necessarily represent those of their affiliated organizations, or those of the publisher, the editors and the reviewers. Any product that may be evaluated in this article, or claim that may be made by its manufacturer, is not guaranteed or endorsed by the publisher.

## Footnotes

^1^Source from http://finance.sina.com.cn/roll/2019-05-27/doc-ihvhiqay1592635.shtml.
